# Avatar-User Bond as Meta-Cognitive Experience: Explicating Identification and Embodiment as Cognitive Fluency

**DOI:** 10.3389/fpsyg.2021.695358

**Published:** 2021-07-08

**Authors:** Young June Sah, Minjin Rheu, Rabindra Ratan

**Affiliations:** ^1^School of Media, Arts, and Science, Sogang University, Seoul, South Korea; ^2^Department of Media and Information, Michigan State University, East Lansing, MI, United States

**Keywords:** avatar-user bond, Proteus effect, meta-cognitive experience, identification, embodiment, cognitive fluency

## Abstract

Scholars have not reached an agreement on a theoretical foundation that underlies the psychological effects of avatar use on users. One group of scholars focuses on the perceptual nature of avatar use, proposing that perceiving the self-being represented by a virtual representation leads to the effects (i.e., Proteus effect). Another group suggests that social traits in avatars prime users causing them to behave in accordance with the social traits (i.e., priming effects). We combine these two theoretical explanations and present an alternative approach, hinging on a concept of *meta-cognitive experience*. The psychological mechanism of the avatar-user bond is explicated in terms of *cognitive fluency*, a type of meta-cognitive experience reflecting an awareness of how readily or easily information is processed. Under this explication, two concepts related to avatar-user bond, identification and embodiment, are understood as the meta-cognitive experience of cognitive fluency at the level of one’s identity and physical body, respectively. Existing empirical evidence on avatar effects is revisited to explore how this new theoretical framework can be applied.

## Introduction

Avatars, defined as virtual representations of selves in artificial and mediated environments, are commonly used in digital worlds, such as video games, virtual environments, and social media. Given the ubiquity of mediated environments—and thus avatars—in our daily lives, there is a potential to harness avatars toward positive outcomes in different contexts, such as promoting healthy behaviors (Fox and Bailenson, 2009) and educational achievement (Ratan et al., 2016). Supporting this idea, the previous studies reported that people, after using avatars, behave in ways consistent with their avatars’ characteristics, a phenomenon known as the Proteus effect (Yee and Bailenson, 2007; Fox and Bailenson, 2009; Fox et al., 2009; Peña et al., 2009).

While empirical evidence supporting the Proteus effect has been accumulating (Ratan et al., 2020), scholars have not reached an agreement on a theoretical foundation for the phenomenon. Yee and Bailenson (2007), who introduced Proteus effect to the field, explained the phenomenon using self-perception theory (Bem, 1972). Peña et al. (2009) employed priming as their theoretical (Chartrand and Bargh, 1999) and methodological foundation (Page and Scheidt, 1971), when investigating whether an avatar with different social characteristics cause users to show psychological and behavioral responses consistent with the avatar’s characteristics. In contrast to the priming account, which assumes the direct, automatic effect of the avatar use, Yee and Bailenson (2009) articulated the importance of the subjective experience in embodying an avatar (i.e., feeling of embodiment) in generating the Proteus effect, proposing that the Proteus effect is qualitatively distinctive from the priming effect, which posits that mere exposure to a visual stimulus elicits automatic psychological and behavioral responses.

Acknowledging that these lines of the literature have not been unified into a single framework, the current study proposes a theoretical foundation that embraces and enhances the previous theoretical approaches to explain the phenomenon. This new approach builds on research in social cognition, incorporating the experiential nature of priming processes using the concept of meta-cognitive experience (Schwarz, 2004). Schwarz (2004) defined meta-cognitive experience as the subjective experience emerging through the processing of information. In particular, the current study hinges on the idea of cognitive fluency, a meta-cognitive experience regarding how readily or easily information is processed (Schwarz, 2004). We propose that cognitive fluency aptly describes the relevant subjective experience of using an avatar, as such experience in a virtual environment speaks to how readily users incorporate different types of information from an avatar into their own self-concept (e.g., self-presence; Ratan, 2012).

To demonstrate the usefulness of this theoretical foundation, we apply it to two well-defined subjective experiences of avatar use in the field of communication—identification and embodiment—and re-conceptualize these constructs under our theoretical framework of cognitive fluency. In particular, we explicate identification as the subjective experience emerging from the fluent cognitive processing of identity-related cues, and embodiment as subjective experience of fluently exerting control over an avatar’s movement. Thus, our prediction is that a user would report greater identification with their avatar when they feel that identity-related information from their avatar is easy to process, and greater embodiment when they feel that the avatar can be easily controlled. Furthermore, we predict that these two distinctive cognitive fluencies simultaneously lead to the Proteus effect, resulting in an assimilation of users’ behaviors with the social characteristics inferable from an avatar.

## Related Work

### Experiential Aspect of the Proteus Effect

The Proteus effect is observed when users of an avatar in a video game or virtual environment assimilate their cognitive states to those assumed from their avatar. The account of the Proteus effect by Yee and Bailenson (2007) originates from self-perception theory (Bem, 1972) and deindividuation theory (Johnson and Downing, 1979). Self-perception theory states that people recognize what other people expect of their behaviors, and then comply to such expectations (Bem, 1972). While early studies of self-perception assumed the importance of presence of others in inducing behavioral confirmation (Snyder et al., 1977), later studies suggested that individuals behave in a way that other people would expect them to behave and adhere to the assumed identity, even if there are no other people holding such expectations at the time of the behavior (Bem, 1972). Thus, avatar users, being reminded of social traits of the avatar, behave in accordance with the behavioral expectations assumed from the social traits of their avatars, despite the absence of others anticipating such behaviors. This tendency of self-perception becomes particularly salient in a deindividuated context, as being deindividuated and indistinguishable from others renders people susceptible to external behavioral or identity cues, such as group identity (Johnson and Downing, 1979). Considering that people are often anonymized and thus deindividuated in virtual environments, it is reasonable to predict that individuals are more likely to adopt the identity given by an avatar and conform to the social expectations associated with the avatar.

In contrast to the identity-based explanation for the Proteus effect, Peña et al. (2009) propose a direct and automatic process of the avatar effect. Their argument is based on priming theory (Bargh, 1994; Higgins, 1996), proposing that human cognition and behaviors are governed by the automatic spreading of the activated knowledge. According to priming theory, exposing individuals to a stimulus with a social trait lead to activation of associated concepts in their mind. The activated concept is more readily accessible than other concepts, and thus easily utilized in one’s cognitive and behavioral processes, resulting in the behavioral assimilation (Loersch and Payne, 2012).

Note that this line of argument implies that the Proteus effect does not rely on higher-level thought processes in generating the behavioral and cognitive effect of avatar use, such as reflection on, “what type of person other people believe I am,” or “how I should behave to be in line with my current identity,” all of which is often presumed in the self-perception account for the Proteus effect. The priming account rather relies on a simple mechanism of the automatic spreading of the activated concept. This account presents a succinct, versatile explanation of the psychological mechanisms underlying a wide range of human behavioral and cognitive processes.

Despite the prevalence of the priming account in explaining human cognition and behaviors in the psychology literature, the application of the theory to the Proteus effect has been questioned. That is, the priming account does not sufficiently address the role of subjective experience of avatar use, often generated by the immersiveness of advanced media technologies (e.g., virtual environment). Indeed, in the research tradition established in social cognition, studies focus on examining how an activated concept exerts influence, not how different types of priming stimuli induce variations in the strength of their influence. This lack of incorporation of the subjective experience into the theoretical framework hinders research on how the advancement of communication technologies enhance (or deteriorate) the subjective experience of avatar use.

Yet, updates in theories of social cognition integrate subjective experience into priming theory. The active-self account (Wheeler et al., 2007, 2014), for example, proposes that the priming effect of a social identity leads to a greater assimilating effect because of an increased association between the priming target and the individual’s self-concept. Self-concept is knowledge of self and can be activated or deactivated by external stimuli (Markus and Nurius, 1987; Markus and Wurf, 1987). A priming target activates the knowledge of the target, but it also invokes compatible self-knowledge or introduces new knowledge to one’s own self-concept. Along with the activated (or updated) self-knowledge, the priming target causes individuals to show greater cognitive and behavioral consistency with the social characteristics of the target. For example, individuals reading a story of an achieving protagonist may become reminded of such a moment in their life (i.e., existing self-concept activated) or feel as if the experience of achievement is their own (i.e., self-concept updated) and thereby show cognitive and emotional responses of achievement.

This active-self account can be formulated as a meta-cognitive experience that relates to the priming account. Meta-cognition, cognition of cognition, refers to individuals’ subjective experiences of processing information. Examples of meta-cognition include the feeling of doing (i.e., sense of agency), feeling of being right, and feeling of knowing. Subjective experiences of feeling of knowing emerge into consciousness, for example, when individuals try to retrieve a certain piece of information from their memory (e.g., encountering a person you think you know). Regardless of whether or not they succeed in the information retrieval, people may experience the processing as either easy or difficult. Such meta-cognitive experience is often understood as cognitive fluency, as the extent to which information is processed fluently (i.e., easily) influences the subjective experience of information processing. Subjective experience is considered a conscious process although individuals might not be aware of the reason for the experience or the source of the experience, whereas cognition is not necessarily a conscious process (e.g., subliminal priming).

The meta-cognitive experience, we propose, appropriately captures the subjective experience of being represented by an avatar in the Proteus effect and thus presents a comprehensive theoretical framework by embracing the experiential aspect of the Proteus effect into the priming account. This theoretical framework re-conceptualizes the immersive experience of using an avatar as cognitive fluency in processing avatar-related information. When people fluently process such avatar information as being associated with their self-knowledge, they experience the avatar as a component of their self-concept in that moment.

### Two Layers of Avatar-User Bond as Cognitive Fluency

Avatar users process avatar information in different layers (Ratan, 2012). In particular, the previous studies on the subjective experience of using avatars propose two routes to the being-an-avatar state: identification (Cohen, 2001; Klimmt et al., 2009) and embodiment (Biocca, 1997; Fox et al., 2013; Ratan and Sah, 2015). To address these distinctive experiences of avatar use, two types of cognitive fluency, one at the identity level and the other at the body-control level, were examined as socio-cognitive origins of the being-an-avatar experience. Our theoretical framework proposes that identity-level fluency leads to subjective experience of identification, and control-level fluency leads to the subjective experience of embodiment.

#### Identification as Cognitive Fluency

Identification has been discussed in many forms of media, from books (Cohen, 2001) to digital games (Van Looy et al., 2012). Particularly due to their technical potentials for presenting realistic graphics and interactivity, digital games have been a venue for examining media users’ identification with their avatars (Konijn et al., 2007; Christy and Fox, 2016). Previous studies on identification explicated the concept from the socio-psychological perspective. That is, media users simulate cognitive and emotional states of a character portrayed in a medium, and experience being identified with the character (Oatley, 1999; Cohen, 2001). In a theoretical development of identification with a video game avatar, Klimmt et al. (2009) clarified the concept as changes in individuals’ understanding of self, for example, who I am and how I should behave in this situation, as a result of avatar use in the video game. Using terms from social psychology, this explanation conceptualizes identification as a shift of media users’ self-concept, by adopting parts or whole of characteristics of an avatar (Klimmt et al., 2009).

The alteration of self-perception proposed by Klimmt et al. (2009) can be understood as activation of a self-concept. What differentiates our framework of meta-cognition from the socio-psychological definition of identification is that self-knowledge activation accompanies the *subjective experience* of activating self-knowledge. Note that prior studies on identification from the socio-psychological perspective did not make a clear distinction between activation of self-concept and subjective experience of the activation. For example, Oatley (1999) referred to identification as an individual’s mental state assimilated with that of a character (i.e., activation of self-concept), but Oatley’s measurement in fact taps a conscious process being integrated with a media character, or using terms we propose, subjective experience of the activation. Also, Klimmt et al. (2010) assumed that self-concept shift and the subjective experience of the shift are the same when they suggested two different ways of measuring self-perception shifting, implicit (e.g., using an Implicit Association Test) and explicit measurements (i.e., using self-reported items). We propose that the implicit and explicit measures distinctively assess the shift of self-concept and subjective experience of the fluent shift, respectively. Note that Klimmt et al. (2009) did not find a significant correlation between the two measures, indicating that subjective experience of knowledge activation is not always dependent on the activation of knowledge itself.

Indeed, the activation of self-perception is not necessarily experienced at a conscious level. Classic social identity literature using a minimal group paradigm (Diehl, 1990) and subliminal priming studies (Bar and Biederman, 1998) did not presume that identity shift includes a conscious process of the priming stimulus. Rather, the shift of self-perception in the minimal group paradigm is assumed to occur at the unconscious level: Self-perception of being a group member is self-knowledge chronically available and even subtle cues in the minimal group paradigm can trigger such activation without conscious experience of shifting self-perception. In contrast to knowledge activation either being a conscious or unconscious process, subjective experience of the self-perception shift is surely a conscious experience, as indicated by the modifier, *subjective*. One might not be aware of the source of the subjective experience, but they should experience the feeling at the conscious level.

Previous studies have examined determinants of the subjective experience of avatar identification. They include design-related factors, such as narratives in a video game (Lee et al., 2006), realistic visual presentations (Slater et al., 2008), similarity in avatar’s appearance (Trepte and Reinecke, 2010), and user trait variables, such as transportability (Christy and Fox, 2016) and interdependent self-construal (Jin and Park, 2009). Identification can be also intensified by contextual factors, such as the amount of time spent playing a video game (Song and Fox, 2016) or customizing an avatar (Turkay and Kinzer, 2014). Note that these components are related to the extent to which participants process information fluently. Design factors, such as narratives, realistic visual graphics, and appearance similarity, facilitate users’ fluent incorporation of identity cues from an avatar. Individual trait variables, such as transportability and interdependent self-construals, can be considered cognitive abilities to process avatar cues as their own. Further, contextual factors, such as amount of time spent playing a video game and customizing an avatar, help avatar users feel familiar with the identity cues of an avatar and thereby make them easy to process. Thus, one might argue that identification is facilitated by fluent processing of identity cues.

#### Embodiment as Cognitive Fluency

Embodiment is also often discussed in the field of communication in relation to subjective experience of using an avatar. While the term has been used to denote different meanings in the literature (for example, Biocca, 1997; Haans and IJsselsteijn, 2012; Costa et al., 2013; Fox et al., 2013; Kim et al., 2014), one early definition described embodiment as a media user’s feeling of body integration with a computer interface in a virtual environment (Biocca, 1997). Biocca (1997) suggested that user’s body and an immersive medium (e.g., virtual environment) become increasingly integrated as technological advancement connect the user’s sensory channels to computer systems’ outputs, while motor controls become linked to systems’ inputs. Biocca’s observation emphasizes the body-level connection between users and the medium through integrating users’ sensory-motor systems and a medium’s input/output channels. The emphasis on the sensorimotor integration with an avatar in embodiment can also be found in a work by Ratan (2012), which suggests different layers of avatar-player connection and proposes that users often experience a connection at a body-schema level of integration. According to this explication, embodiment is a body-level connection (i.e., proto self-presence), distinctive from emotion- (i.e., core self-presence), and identity-level connections (i.e., extended self-presence) with avatars.

While this line of the literature on embodiment did not elucidate whether embodiment refers to coupling between users’ motor-sensory systems and computer systems or subjective experiences obtained from the coupling, such embodiment has been often measured by asking to what extent users feel their body is extended into an artificial object, assessing the experiential nature of embodiment. In line with this tradition, we define embodiment as a meta-cognitive experience of owning an artificial object as a user’s body part, which results from a user’s sensory-motor system integration with the artificial object.

Being in a state of owning a body through sensory-motor integration and the subjective experience of owning a body is hardly distinguishable under normal situations, but occasionally they are decoupled. For example, people mistakenly believe that they have a hand and feel pain in the hand, even though they do not actually possess the hand. Known as phantom limb pain (Subedi and Grossberg, 2011), patients suffering from it have a subjective feeling of owning the body part, but they are not in a state of owing it. Also, studies on rubber hand illusion illustrate that people can be induced to feel a fake rubber hand as their own (Botvinick and Cohen, 1998). This illusion occurs when tactile stimulation is simultaneously applied to a rubber hand placed in front of an individual alongside their hidden own hand. By integrating the visual information of a rubber hand being stimulated and sensory information feeling the stimulation from the invisible actual hand, people have the subjective experience of owning the rubber hand and show proprioceptive alteration in response to stimuli applied to the rubber hand (Botvinick and Cohen, 1998). In this case of the rubber hand illusion, being in state of owning a hand and subjective experience of owning hand are dissociated in two ways: People own their actual hand thus can move their actual hand volitionally, but they lose subjective experience of owning their actual hand, and they subjectively experience ownership of the rubber hand despite not truly owning it.

This line of argumentation can be applied to avatar users’ experience of embodiment. When a user’s sensory-motor system is fluently integrated with an avatar in a virtual environment (e.g., natural control of an avatar in a video game), users of the avatar may experience embodiment or the subjective experience that the avatar’s body is their own body. Note that technological advancements in digital game interfaces, such as the Nintendo Wii or Xbox Kinect, enable fluent integration by synchronizing players’ inputs and outputs using, for example, gestural inputs corresponding with on-screen visual updates. We further propose that even controllers with simple symbolic mapping (e.g., joysticks and keypads) may induce a meta-experience of owing the avatar’s body as players become accustomed to controlling the avatar and thereby are able to fluently process sensory-motor information. Supporting our proposal, the previous studies show that using a simple tool (e.g., rake) can be integrated into (monkey’s) body schema (Maravita and Iriki, 2004) and an alien body part presented in a virtual environment can be harnessed by users after repeated uses (Won et al., 2015). Thus, the fluent control of an avatar may lead to the subjective experience of embodiment.

## Discussion

The current paper proposes a novel approach of cognitive fluency to the user-avatar bond. Building upon the concept of meta-cognitive experience, we attempt to integrate the two existing perspectives, i.e., the Proteus effect and priming effect. The Proteus effect focuses on the experiential nature of avatar use and provides a useful theoretical framework to investigate how advanced communication technologies (e.g., immersive virtual environments) enable users to feel subjective experiences of being integrated with the avatar. In contrast, the priming account of avatar effects provides a theoretical foundation regarding how the avatar may lead to automatic and unconscious effects on users’ cognitive and behavioral responses, uncovering unique aspects of the user-avatar bond.

By combining these two approaches, we re-conceptualize the user-avatar bond as a function of cognitive fluency, a meta-cognitive experience of smooth information processing regarding an avatar in a virtual or mediated environment. We propose that different aspects of the avatar-user bond can lead to fluent cognitive process, focusing specifically on identification and embodiment. Our proposal provides a theoretical implication regarding user-avatar bond research. That is, the two theoretical accounts of the Proteus effect and priming effect are not in conflict but complementary to each other when considering that the experiential aspect and the automatic nature of the user-avatar bond are interdependent.

The cognitive-fluency account allows us to propose unique predictions regarding factors influencing the user-avatar bond. For example, we suggest that social characteristics of an avatar (e.g., an inventor avatar) may lead to an expected result of priming (i.e., more creative behavior; Guegan et al., 2016), but the effect can be moderated by the subjective experience of fluent processing of the avatar information. Those who have spent more time customizing an avatar might process the information of using the avatar more fluently, meaning that they adopt the avatar identity more readily, yielding a stronger effect of using the avatar. By employing a proper research design, one may disentangle the unconscious effect of avatar use and the effect of subjective experience of cognitive fluency.

While the current study presents meaningful extensions of the theoretical foundation of avatar effects, future work should be conducted to empirically test the propositions and explore unasked questions. Drawing on our articulation of the cognitive fluency as the user-avatar bond relating to identification and embodiment, one may ask what factors influence the different types of cognitive fluency. One possible factor is similarity of an avatar to its user, which we expect to lead to fluent processing of the identity and thus enhance identification. Thus, it can be predicted that the more similar an avatar is to its user in internal (e.g., personality) and external aspects (e.g., appearance), the greater cognitive fluency in processing cues of the avatar, resulting in a stronger Proteus effect. Also, we suspect that realistic mapping in controlling an avatar may lead to fluent processing of the control, which in turn can facilitate the sense of embodiment with the avatar.

Further, the future research may explore how the meta-cognitive experience of identity and control interplay to contribute to the user-avatar bond. The current study argues independent paths of identity and control fluency to the subjective experience of identification and embodiment, respectively. Yet, one may expect that cognitive fluency at the identity and control level may produce simultaneous effects on the user-avatar bond. These expectations should be empirically tested with measures assessing the level of cognitive fluency. As not much empirical research has been conducted using the cognitive fluency as the theoretical framework, the future studies can explore potential measures and test their validity.

One may also attempt to extend the theoretical framework to include additional avatar-experiential constructs other than identification and embodiment. For example, presence (Lee, 2004), immersion (McGloin et al., 2013), and flow (Cowley et al., 2008) are often employed to explain the experience of using an avatar in a virtual or mediated environment. Despite the nuanced differences these concepts manifest, the future research should investigate how they are related to the meta-cognitive experience and cognitive fluency of avatar use. Considering that cognitive fluency is determined immediately as the information is processed, cognitive fluency may influence also other avatar-related constructs. Yet, immersion and flow may influence the cognitive fluency as they relate to avatar users’ mental states in processing avatar information. Future studies may work toward explicating these concepts and their relationships. Such exploration may extend our understanding on how video game, virtual world, and social media users develop bonds with their avatars.

## Author Contributions

YS, MR, and RR developed the idea. YS wrote the manuscript. MR and RR reviewed the manuscript. All authors contributed to the article and approved the submitted version.

### Conflict of Interest

The authors declare that the research was conducted in the absence of any commercial or financial relationships that could be construed as a potential conflict of interest.
